# Structural Patterns of Rhamnogalacturonans Modulating Hsp-27 Expression in Cultured Human Keratinocytes 

**DOI:** 10.3390/molecules13051207

**Published:** 2008-05-27

**Authors:** Vincent Gloaguen, Pierre Krausz, Véronique Brudieux, Brigitte Closs, Yves Leroy, Yann Guerardel

**Affiliations:** 1 Laboratoire de Chimie des Substances Naturelles (UPRES-EA 1069), Université de Limoges, Faculté des Sciences et Techniques, 123 rue A. Thomas, 87060 Limoges, France; 2 R&D Department, SILAB, ZI de la Nau, 19240 Saint-Viance, France; 3 Unité de Glycobiologie Structurale, UMR-CNRS 8576, Université de Lille, F-59655, France

**Keywords:** Pectin, rhamnogalacturonan, structure, keratinocyte differentiation, structure‑activity relationships

## Abstract

Polysaccharide extracts were obtained from chestnut bran (*Castanea sativa*), grape marc (*Vitis vinifera*) and apple marc (*Malus* spp.) and fractionated by size exclusion chromatography after endopolygalacturonase degradation. Compositional and linkage analyses by GC and GC-MS showed the characteristic rhamnogalacturonan structure with specific arabinan (apple marc) and type II arabinogalactan (chestnut bran, grape marc) side chains. Type II arabinogalactan rhamnogalacturonan from chestnut bran significantly stimulated the *in vitro* differentiation of human keratinocytes, giving evidence of a tight structure-function relationship. This molecule comprises short and ramified 3- and 3,6-β-d-galactan and 5- and 3,5-α-l-arabinan side chains, but also contains significant amounts of *t‑*Xyl and 4‑Xyl with a characteristic 2:1 ratio. Enzymatic hydrolysis of this polysaccharide produced fragments of lower molecular weight with unchanged xylose content which conserved the same ability to stimulate human keratinocyte differentiation. It could be then speculated that dimeric xylosyl-xylose and/or longer oligomeric xylose side chains attached to a galacturonan and closely associated to hairy rhamno-galacturonan domains are essential patterns that could determine the biological activity of pectins.

## Introduction

Plant cell walls are known as potential sources of pharmacologically active polysaccharides [1,2]. These polysaccharides can be obtained from a wide variety of botanical sources and some of them are currently used for their numerous effects on skin [3,4]. Better than simple excipients in dermatological or cosmetic formulations of gels, ointments or lotions, they are now recognized as active entities. As an example, plant polysaccharides promote the proliferation and differentiation of human epithelial cells (keratinocytes and fibroblasts) [5,6]. Among plant polysaccharides, pectins have long been used by traditional pharmacopoeias [7]; these anionic polysaccharides have recently attracted a lot of interest and been subjected to extensive structural studies. Like other categories of plant poly-saccharides, pectins are polydisperse macromolecules with substantial heterogeneity in terms of molecular mass as well as chemical structure. Their composition is strongly dependent on their origin, their localization within the plant and is also influenced by the extraction procedures used. Pectins consist of a backbone, mainly composed of galacturonic acid and a smaller amount of rhamnose; these molecules also include arabinan and/or arabinogalactan side chains, primarily composed of arabinose and galactose, linked to the carbon 4 of rhamnose units [7], hence the discrimination between two structurally distinct entities: “smooth regions” and “hairy regions”, containing homogalacturonan (HG) or rhamnogalacturonan (RG) respectively. It has also been reported that homogalacturonans can be substituted, often with single xylose β-(1-3) linked to GalA residues to form xylogalacturonans (XG).

The structure-function relationships of pectins are currently under investigation, but definite data are still lacking about the actual bioactive patterns of pectic polymers [7,8]. Bio-activity of pectins has been frequently correlated with their acidity, even though this characteristic is not an absolute requirement [6]. On the other hand, increased degrees of polymerization as well as extensive branching and ramification seem to enhance their biological activities [8]. Rhamnogalacturonan from *Hibiscus sabdariffa* was shown to stimulate the proliferation and differentiation of human keratinocytes [9]. Deters and coworkers recently suggested that terminal arabinose residues of type I RG arabinogalactan side chains [10], as well as rhamnose content [11], could justify the observed bio-activity of pectins. More precisely, rhamnogalacturonans were shown to bind outer membrane receptors, triggering a signal transduction cascade and ultimately nuclear activation [10]. 

In our previous works [12,13] we characterized the *in vitro* and *in vivo* biological activities of a rhamnogalacturonan isolated from chestnut bran (*Castanea sativa*). The results obtained demonstrated its stimulating effect on human keratinocyte differentiation. Nevertheless, the question of the chemical origin of such a bioactivity remained unanswered. For this reason we undertook this study with the aim of identifying the chemical part of the molecule responsible for its bioactivity. To this end, the structure and biological activities of rhamnogalacturonan with: *i)* type II arabinogalactan‑rich side chains isolated from chestnut bran (*Castanea sativa*), grape marc (*Vitis vinifera*) and *ii)* arabinan rich side chains isolated from apple marc (*Malus* spp) were compared, giving evidence of a tight structure‑function relationship. 

## Results and Discussion

Raw polysaccharides (crude extracts) with a carbohydrate content >98% and less than 1.5% residual impurities (protein and polyphenols) were isolated in yields varying from 6.8 % (apple marc) to 46.4% (chestnut bran) (Table 1).

**Table 1:** Yield of polysaccharide extraction from raw materials and characteristic uronic acid content of the extracts. UA = Uronic Acid.

Plant materials and fractions	Yield of extraction (W/W Total Sugar / Raw Material)	%[UA]
**Chestnut bran**		
*Crude extract*	*46.4*	*8.4*
FI	0.6	50
FII	1.3	80
FIII	44.5	0
**Apple marc**		
*Crude extract*	*6.8*	*69.5*
FI	3.3	47.1
FII	2.7	86.8
FIII	0.8	8.5
**Grape marc**		
*Crude extract*	*24.0*	*36.9*
FI	8.0	42.9
FII	6.9	54.6
FIII	9.1	34.0

Uronic acid contents of the aqueous extracts ranged between 8.4% (chestnut bran) and 69.5% (apple marc). Except for chestnut bran extract, whose composition was dominated by Glc as a result of starch contamination, Rha, GalA, Ara and Gal were the characteristic monosaccharides units identified in the polysaccharidic fraction, confirming the typical rhamnogalacturonic nature of these extracts (Table 2). Hydrolysis by endoPG, followed by chromatographic fractionation on Biogel P6 led to the separation of acidic polysaccharidic (FI), acidic oligosaccharidic (FII) and neutral di- and monosaccharidic (FIII) fractions. Table 2 shows their respective carbohydrate compositions. With high amounts of GalA, Rha, Ara and Gal, all three of the FI subfractions present the typical compositions of pectic rhamnogalacturonans. This conclusion is confirmed by GC-MS linkage analysis of methylated monosaccharide derivatives identified as the characteristic rhamnogalacturonan side chains (Table 3). With high contents of *t*, 5- and 3,5-Ara, FI apple marc fraction is typical of a rhamnogalacturonan with arabinan side chains (Figure 2); this is in full agreement with earlier findings [14]. In addition, the presence of 5- and 3,5-Ara and 3- and 3,6-Gal units in grape marc rhamnogalacturonans is characteristic of type II arabinogalactan side chains (AG-II); again, these data are in full agreement with earlier findings [15]. 

**Table 2:** Monosaccharide composition (molar ratio) of extracts; t = trace.

Plant materials and fractions	Ara	Rha	Xyl	Man	Gal	Gal A	Glc	Glc A
**Chestnut bran**								
*Crude extract*	*t*	*t*	*0*	*0*	*4.0*	*t*	*96.0*	*0*
FI	14.6	16.6	9.4	1.4	15.0	38.2	2.2	2.5
FII	6.4	3.6	10.2	0.8	1.7	65.9	11.3	0
FIII	0	0	0	0	0	0	100	0
**Apple marc **								
*Crude extract*	*36.4*	*6.3*	*0*	*0*	*6.8*	*50.5*	*0*	*0*
FI	29.1	6.0	2.5	2.3	3.9	53	1.4	1.7
FII	10.0	1.9	1.2	0	0.5	81.6	0	4.8
FIII	34.8	5.9	9.7	0.7	20.7	3.5	24.7	0
**Grape marc**								
*Crude extract*	*26.2*	*9.1*	*5.7*	*20.2*	*13.0*	*21.2*	*4.5*	*0*
FI	24.5	11.2	5.5	9.4	15.6	29.0	1.3	3.4
FII	18.9	7.8	7.1	7.0	8.9	41.9	0	8.4
FIII	12.9	3.9	4.1	44.2	14.1	t	20.8	0

**Table 3:** Typical side chains glycosyl-linkage of FI and FII fractions isolated from chestnut bran, apple and grape marc rhamnogalacturonans. Data indicate the respective % of peak areas.

Carbohydrates	Chestnut bran		Apple marc	Grape marc
	FI	*FII*	FI	FI
**Arabinose**				
*t*-Ara	14.5	*16.9*	35.0	28.0
5-Ara	14.5	*15.2*	23.2	12.7
3,5-Ara	2.7	*11.1*	14.6	8.5
**Galactose**				
*t*-Gal	13.5	*10.7*	5.8	6.7
3-Gal	9.3	*2.3*	3.1	11.6
4-Gal	13.4	*10.8*	10.9	4.3
6-Gal	5.7	*0*	1.1	3.7
3,6-Gal	12.9	*3.3*	3.7	20.5
4,6-Gal	0	*4.7*	0	0
**Xylose**				
*t*-Xyl	8.6	*18.7*	2.6	4.0
4-Xyl	4.9	*6.3*	0	0

**Figure 1:**
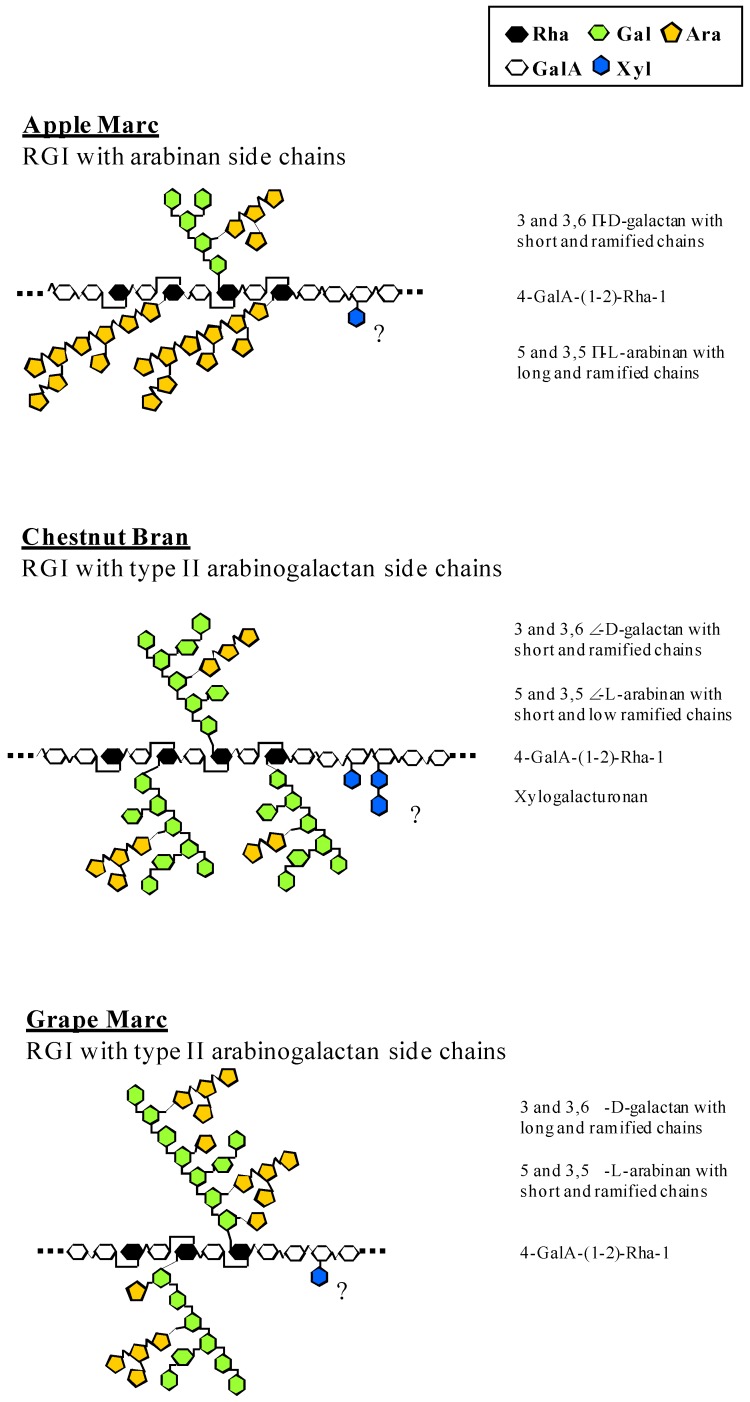
Hypothetical structures of the rhamnogalacturonans extracted from chestnut bran and apple and grape marcs.

Such distribution could also be observed in chestnut bran rhamnogalacturonan side chains. Lastly, short 4-linked galactans could be identified in both chestnut bran and grape marc rhamno-galacturonans, although in smaller amounts. In the case of grape marc, higher amounts of 3 and 3,6-Gal and lower amounts of *t*-Gal suggest the presence of longer galactan chains (Figure 1). Conversely, with a higher amount of t‑Gal and a lower amount of 3,6-Gal, arabinogalactan from chestnut bran rhamnogalacturonan appears as a more condensed structure (Figure 1). Lastly, with the highest amounts of GalA residues (up to 81.6% molar ratio in apple marc), FII rhamnogalacturonan subfractions could be considered as the most acidic fractions.

Cytotoxic properties of crude rhamnogalacturonans were studied in a preliminary experiment (Table 4). A concentration of 0.02% (w/v) appears, for the three extracts, as the highest concentration that did not induce any cytotoxic effect on cultured human keratinocytes. 

**Table 4:** Cytotoxic properties of rhamnogalacturonan crude extracts. Cellular viability is expressed in %. Co = Control.

Concentrations (%W/V)	Co	0.004	0.01	0.02	0.04	0.1	0.2	0.4
**Chestnut bran**	102	99	98	**100**	117	92	78	60
**Apple marc**	100	123	130	**121**	84	13	10	10
**Grape marc**	103	103	99	**100**	50	35	20	15

**Figure 2:**
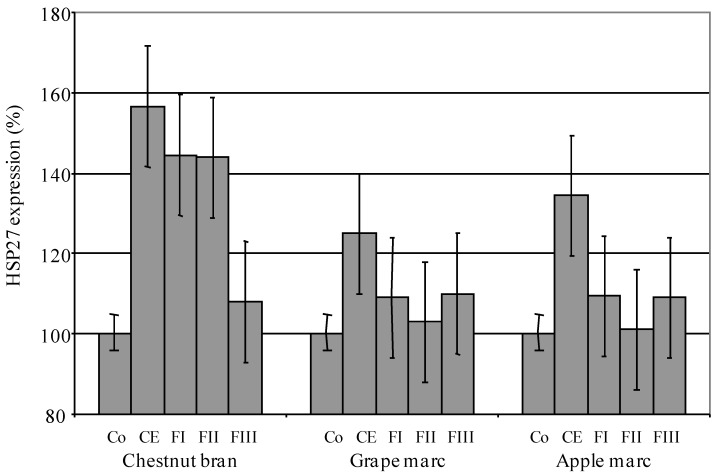
Keratinocyte HSP27 expression promoting effects induced by rhamno-galacturonan crude extracts (CE) and endo-PG degraded rhamnogalacturonan FI, FII and FIII subfractions. Co = control, CE = 0.02% (w/v), [FI] + [FII] + [FIII] = 0.02 % (w/v).

This concentration was then selected as the test dose to study the effects of rhamnogalacturonan crude extracts (CE) and endo-PG degraded thamnogalacturonan FI, FII and FII subfractions on human keratinocyte differentiation. With an increase of human keratinocyte HSP27 expression ranging from 125 % (grape marc) to 156% (chestnut bran), the three rhamnogalacturonan crude extracts tested significantly stimulated cellular differentiation (Figure 2). Fractionation of grape and apple marc crude extracts on Biogel P6 led to a significant decrease of their HSP27 stimulating power, although polysaccharidic FI as well as oligosaccharidic FII subfractions derived from endoPG hydrolysis of chestnut bran rhamnogalacturonan crude extract, conserved the same level of activity. 

Focusing on the dermatological activities of plant-derived polysaccharides, we recently characterized the stimulating effect of chestnut bran rhamnogalacturonan on human keratinocyte differentiation [12]. Since arabinan and arabinogalactan chains covalently linked to RG-I structures have been associated with strong biological activities [3] and especially keratinocyte proliferation and differentiation [6,9,10,11], we wanted to investigate the structural characteristics of three extracts - *an arabinan and two AG-II type RG-I polysaccharides* - with the aim to characterize the variability of their properties and then to identify the structural origin of their bioactivity. Mr 27,000 heat shock protein (HSP-27) is a member of the small heat shock protein family. Kindas and Trautinger [16] and Jonack *et al.* [17] provided evidence that HSP-27 was accumulated in a differentiation-dependent manner in normal human keratinocytes grown under conditions inducing terminal differentiation. Heat shock protein HSP-27 was then considered as a good differentiation marker of primary NHK cells. All the crude rhamnogalacturonic extracts tested significantly stimulated keratinocyte differentiation, but their endopolygalacturonase hydrolysis products showed specific properties with this respect. These products, identified as FI, FII and FIII Biogel P6 fractions, represented the poly-, oligo- and di/monosaccharidic fractions respectively (Figure 2). Thus, the size of these polysaccharides, as well as their characteristic monosaccharidic composition, seems to modulate their biological activity. These conclusions are supported by several reports [6,18,19,20] in which similar structure/function relationships have been observed. In their review dedicated to bioactive polysaccharides [7], Paulsen and Barsett pointed out that most of these molecules presented a typical rhamnogalacturonan I backbone. They generally possessed arabinogalactan II side chain, while a few of them were found with arabinogalactan type I chains. Then, it can be ascertained that the structure and the variability of rhamnogalacturonan I side chains are the most important structural features that could modulate the bioactivity of pectins. With its characteristic arabinan side chains (Table 3, Figure 2), the FI RG-I subfraction from apple marc does not stimulate keratinocyte differentiation. This is also true for its acidic FII subfraction. Biological activity of the RGI - FI subfractions obtained from endoPG digestion of grape marc and chestnut bran pectic crude extracts with respectively - 108 and 144% of HSP 27 expression, are quite different though they both present similar AGII side chain composition (Table 3, Figure 2). More interesting is the fact that, in this case, FII subfraction obtained from chestnut bran is the one that preserved the same level of FI keratinocyte stimulating property. Nevertheless, an increase in GalA and a significant decrease in Ara and Rha content could be observed from the FI to the FII chestnut bran fractions. From a structural point of view, such modifications could affect acidity, conformation of the flexible hairy region of the pectins as well as the ratio smooth/hairy regions but, taken alone and according to our results, these variations are not sufficient to explain the variability of the RG-I effects on human keratinocyte physiology. The relatively high amount of xylose must be pointed out. As indicated in the introduction, pectins are a family of complex acid hetero-polysaccharides containing distinct structural domains among which are found substituted galacturonans of the xylogalacturonan type. In this case, backbone GalA is frequently C-3 substituted with monomeric xylose [21,22] and/or, although less frequently, dimeric xylosyl-xylose units [23]. Among the three rhamnogalacturonic FI fractions tested, chestnut bran is the only one that stimulates human keratinocyte differentiation (Figure 1). This fraction comprises both *t-*Xyl and 4-Xyl suggesting - *taking into account the characteristic ratio t-Xyl/4-Xyl = 2* - the presence of a dimeric xylose substitution in an oligoxylogalacturonan motif closely associated to rhamnogalacturonan (Table 3, Figure 2). FII chestnut bran subfraction is also the only one that preserved a similar F1-level of keratinocyte differentiation stimulation. Compared to the FI subfraction, its 4-Xyl content is also unchanged. With *t*-Xyl as the unique kind of xylose substitution (Table 3, Figure 2), neither the FI nor the FII subfractions obtained from grape or apple marc actually stimulate keratinocyte differentiation. Thus xylosyl dimeric chains appear as a key marker of pectin bioactivity. A similar hypothesis could be supported by the observed properties of two acidic pectic polysaccharides from *Glinus oppositifolius* [24] which are characterized by dose-dependent complement-binding activities. Of the two extracts tested, the one that induced the highest level of chemotaxis in T and NK cells is also the one that presented characteristic *t-*Xyl and 4-Xyl contents. Recently, similar findings have been obtained with a polysaccharide extracted from *Biophytum petersianum* K. [25]. 

## Conclusions

In summary, the relatively high amount of xylose observed in chestnut bran rhamnogalacturonan is not common in pectins, but has been observed in various plants and is often associated with the xylogalacturonan (XG) regions. The exact location of xylose in pectins is still the subject of controversial debates in the literature. XG is considered either as a part of the pectic backbone or as belonging to side chains [23]. Normally linked to the GalA residues, some xylosyl residues were recently found directly linked to *O*-4 of rhamnose residues [26], and thus cannot be considered in a strict sense as belonging to the xylogalacturonan backbone. The presence of a dimeric xylosyl-xylose and/or longer oligoxylose side chains as well as its/their location along the rhamnogalacturonan I backbone could justify the large diversity of biological properties of xylose-containing pectin. 

## Experimental

### Biological material

Chestnut bran (*Castanea sativa*) was commercially obtained as a lyophilized powder from the society Cailleau SA (Chemillé, 49120 France). Apple (*Malus spp*.) and grape (*Vitis vinifera*) marcs were commercially obtained as lyophilized powders from the society Les Tisanniers (Saint Augustin, 19390 France).

### Chemicals and enzymes

All chemicals used were of analytical grade and were purchased from Sigma. Pullulanase (EC3.2.1.41), α-amylase (EC3.2.1.1) and endopolygalacturonase (EC3.2.1.15) used to prepare rhamnogalacturonans from plant material were obtained from Sigma. 

### Isolation, enzymatic hydrolysis and fractionation of rhamnogalacturonans

Rhamnogalacturonans extraction was adapted from a previously described protocol [12]. Crude chestnut bran, apple and grape marcs were poured into hot water (90 °C) and the resulting mixtures centrifuged. Supernatants were freed of starch by α-amylase/pullulanase digestion (0.5% v/v, 1 hour at 55 °C) and then dialyzed against distilled water (Spectrapor MWCO 6‑8 000 Da cut-off). Rhamnogalacturonans were isolated from concentrated supernatants (4:1 v/v) after precipitation by the addition of 3 volumes of ethanol and subjected to endo-α-1,4-polygalacturonase digestion (EndoPG - 0.1% v/v, 2 hours at 37 °C). The mixtures were then brought to boil for 1 min; the coagulated proteins were then removed by centrifugation and the supernatant further fractionated onto Biogel P6 columns (Bio-Rad; 2x80 cm) with water as eluent. According to the characteristic RI Size Exclusion Chromatography detection and their typical Thin Layer Chromatographic separation (data not shown), three fractions were collected: FI, containing *polysaccharides* eluted in the void volume of the column, FII mainly composed of *oligo-uronides and GalA* eluted within the included volume and FIII mainly composed of neutral *mono- and disaccharides* eluted before the total working bed volume. These fractions were freeze-dried prior to chemical analysis and evaluation of their biological properties.

### Composition of polysaccharides

Total carbohydrate was measured by the phenol-sulphuric acid method [27] with Glc as standard; monosaccharides and hexuronic acids were determined by the *m*-hydroxydiphenyl method [28], with GalA as standard uronic acid. Monosaccharide determination was carried out after methanolysis (0.5 M MeOH/HCl, 24 h, 80 °C) by gas-liquid chromatography of pertrimethylsilylated methylglycosides according to Kamerling *et al.* [29], as modified by Montreuil *et al.* [30]. GLC was performed on a Perichrom gas chromatograph fitted with a flame-ionisation detector. A CPSIL-5CB (Chrompack, 0.32 mm x 50 m) capillary column was used with the following temperature programs: 120 °C - 240 °C at 2 °C.min^-1^. Nitrogen was the carrier gas at 0.5 atm. 

### Monosaccharide linkage analysis

Linkage positions were determined by analysis of partially methylated and acetylated reduced derivatives of monosaccharides. Briefly, oligosaccharides were permethylated with butyllithium according to Parente *et al*. [31], hydrolysed, reduced and acetylated, as described by Kim and Carpita [32]. The resulting partially methylated alditol acetates were analysed by GC-MS using a Carlo Erba GC 8000 gas chromatograph equipped with a 25 m x 0.32 mm CP-Sil5 CB Low bleed/MS capillary column, 0.25 μm film phase (Chrompack). Temperature of the Ross injector was 260 °C and samples were analysed using a temperature program starting at 90 °C for 3 min, followed by a temperature ramp of 5 °C min^-1^ to 260 °C. EI mass spectra were obtained using a Finnigan Automass II mass spectrometer. Compounds at each peak were characterised by an interpretation of the characteristic mass spectra and retention times in comparison to standard sugar derivatives.

### Submerged keratinocyte cultures

Normal Human Keratinocytes (NHK) were isolated as primary cells from surgical resection of human skin. Cells were cultured as described by Deters *et al.* [11] and suspended in K-SFM keratinocyte medium (Invitrogen) supplemented with bovine pituitary extract (BPE, Invitrogen) and epidermal growth factor (EGF, Invitrogen). Incubation was performed at 37 °C in a humidified, 5% carbon dioxide atmosphere. 

### Characterization of keratinocyte physiology

#### Keratinocyte viability

Keratinocytes were seeded into 96-well cell plates (9 x 10^3^ cells/well). Cell viability was then evaluated using the MTT microculture tetrazolium assay (Sigma, St. Louis, MO) according to Mosmann [33].

### Keratinocyte differentiation, quantification of the differentiation-specific protein HSP-27

1x10^6^ NHK were plated onto 100 mm cell culture plates and incubated 4 days at 37 °C in a humidified, 5% CO_2_ atmosphere. Pectic extracts were then added at a final concentration of 0.02% (w/v). Cells were harvested after a 4 day contact time. Cell extracts were obtained after cell disruption with a Potter-Elvehjem homogenizer and centrifugation of cell debris. Bicinchoninic acid (BCA) protein assay (Sigma) was carried out for measuring total protein of supernatants. After standardization of protein content, HSP27 was estimated by the dot blotting technique using anti‑HSP27 monoclonal mouse antibodies (NCL-HSP27, Novocastra) [16,17] 
